# Video Gaming in Older People: What Are the Implications for Cognitive Functions?

**DOI:** 10.3390/brainsci14070731

**Published:** 2024-07-21

**Authors:** Liliana Dell’Osso, Benedetta Nardi, Leonardo Massoni, Simone Battaglini, Chiara De Felice, Chiara Bonelli, Stefano Pini, Ivan Mirko Cremone, Barbara Carpita

**Affiliations:** Department of Clinical and Experimental Medicine, University of Pisa, 56126 Pisa, Italy; liliana.dellosso@gmail.com (L.D.); l.massoni@studenti.unipi.it (L.M.); battaglini.simone@gmail.com (S.B.); c.defelice@studenti.unipi.it (C.D.F.); chiarabonelli.95@hotmail.it (C.B.); stefano.pini@unipi.it (S.P.); barbara.carpita@unipi.it (B.C.)

**Keywords:** video games, mild cognitive impairment, elderly, older subjects, cognitive function, fall prevention, psychiatric rehabilitation

## Abstract

Mild cognitive impairment impacts a sizable segment of the older population, and often evolves into dementia within a few years. At this stage, subjects may benefit from non-pharmacological therapies that can delay or stop the progression of the mild cognitive impairment into dementia and are crucial for improvement in the subject’s quality of life, while also being easily accessible and safe for use. Many research studies have shown that a variety of exercises, including cognitive training, have the potential to enhance or optimize cognitive function and general well-being. Recently, many authors have suggested video games as a promising approach for cognitive training and neurorehabilitation in older people, thanks to their increasing motivation and training effects through immersion in stimulating environments. Under this premise, our narrative review’s objective is to discuss and summarize the body of existing material on the role of video games in improving cognitive performance, daily life activities, and depression symptoms in older individuals with different levels of cognitive decline. From the papers reviewed, it emerged that older subjects trained with video games showed a significant improvement in cognitive functions, sleep quality, and psychiatric symptoms, positioning video games as an intriguing and useful tool.

## 1. Introduction

The population of senior citizens suffering from dementia is growing at an exponential rate. Mild cognitive impairment (MCI) is a neurocognitive impairment defined by the Diagnostic and Statistical Manual of Mental Disorders as the presence of cognitive deficits that are not expected for an individual’s age and education level, but which are nonetheless mild enough not to significantly affect basic daily living activities [1,2]. MCI covers both memory and non-memory deficits, and its cause and means for prevention and therapy are still unknown [3]. The onset and course of MCI can serve as a transition period between normal aging and dementia, particularly Alzheimer’s disease. Indeed, about 50% of those who are diagnosed with the ailment go on to develop Alzheimer’s disease within five years [4,5,6]. For this reason, even though MCI may not worsen and may even go away, its diagnosis can be helpful as a marker for other forms of dementia in the early stages [7]. Dementia is an intricate process that involves the interaction of several molecular pathways that impact cellular functions. These pathways ultimately result in the destruction of neural connections, cellular death, gliosis and inflammation, which in turn causes a person to lose cognitive functioning—the ability to think, remember, and reason—to the point where it interferes with their autonomy in day-to-day activities [8,9]. The intensity of dementia varies; at its mildest, it only starts to interfere with a person’s ability to function, while at its most severe, it renders a person totally dependent on others for even the most basic daily functions. Millions of individuals are impacted by dementia, which is more prevalent as people age, but is not a normal aspect of aging [10]. Early preventive interventions are thus critical because they can lead to significant improvements not just in self-reported quality of life, but also in the general state of health and the financial stability of affected individuals. At this stage, patients may benefit from non-pharmacological therapies that can delay or stop the progression of MCI into dementia. Non-pharmacological treatments are defined as “any theoretically based, nonchemical, focused, and reproducible intervention, performed involving the patient, that may offer any substantial benefit” [11]. Non-pharmacological treatments encompass interventions of various natures, such as nutritional therapies, art-oriented therapies, recollection therapy, physical activity, and cognitive training [12]. In addition to being useful in managing clinical symptoms, they are likely to be crucial in both primary and secondary dementia prevention. Non-pharmacological treatments have several benefits, including being widely accepted, having few negative side effects, and being able to be taken both sequentially and simultaneously with pharmaceutical therapies without raising serious issues about interactions. Non-pharmacological treatments can also be used for individuals who are cognitively normal but are at risk of developing dementia, as well as for patients in various clinical phases, from full blown dementia to moderate cognitive impairment [2,13,14,15]. Consequently, these treatments may have a significant effect on quality of life, well-being, and cognition across the course of age-related neurodegenerative illnesses. Exercises including cognitive training have the potential to enhance or optimize cognitive performance and overall quality of life [16,17].

Given their ease of access and high safety, nonpharmacological therapies are now the go-to method for treating and caring for individuals with cognitive impairment. Recent evidence has highlighted the advantages of physical exercise for the brain heath of older individuals [18], even from a neurobiological point of view [19]. Additionally, music therapy, dancing therapy, art therapy, and reading therapy are some of the main approaches employed in subjective cognitive impairment, that is, the subjective experience of cognitive decline, without objective impairment on cognitive assessment [20]. Another non-pharmacological strategy that has been receiving growing interest for older individuals with cognitive decline and MCI is the use of virtual reality, with cognitive functions, instrumental activities in daily life, and neural efficiency being the main domains improved by this intervention [21,22].

Given this framework, video games seem to be a promising approach for cognitive training and neurorehabilitation in older people, taking effect by increasing motivation and training effects through immersion in stimulating environments [15]. A video game is commonly described as “an electronic or computerized game played by manipulating images on a video display or television screen”. A typical video game has an objective, reward, or incentive system; is interactive and/or competitive; and is made with enjoyment (i.e., fun) in mind [23]. Two main categories of video games can be recognized: amusement games and games for cognitive training. While games for entertainment are concentrated on personal enjoyment and pleasure, cognitive training games are created by neuroscientists with the goal of training cognitive skills [24]. In particular, the most common cognitive training games involve puzzle games, including sudoku, crosswords and jigsaw puzzles; memory games, such as matching cards and memory cubes; logic games, like chess and solitaire; and brain teasers. In recent times, this kind of game has been utilized to help older people receive more cognitive stimulation; for instance, during the social isolation that resulted from the COVID-19 pandemic [25]. Indeed, although the use of video games has been canonically associated with adolescence or young adulthood, the most recent findings from epidemiological studies on today’s video game players challenge traditional stereotypes. In fact, it has been estimated that the average player is around 34 years old; that 40% of players belong to the female gender; and that at least as many as 26% are over 50 years old [26]. Video games have gained increasing attention regarding their hypothetical use in health-promoting programs, proving useful in promoting physical activity, health education, self-esteem, and various spirometric measures. Furthermore, their effectiveness and usefulness in cognitive training programs has also been highlighted. For cognitive training interventions to be beneficial, the activities must be carried out over a substantial amount of time without losing motivation, and cognitive computer games can be used to pique this interest [27]. The use of video games in cognitive training in older people is made possible by technological advancements; this method increases participant motivation and lowers dropout rates by making exercises more enjoyable and engaging. It also has the added benefit of being more widely available to the general public [28,29]. In particular, the application of computer software, video games, and virtual reality platforms has grown in popularity in the fields of sports, education, and health due to the rapid advancement of science and technology and their widespread accessibility [30].

Moreover, different measures, including time of reaction, response precision, precise duration of practice, and physiological indicators, like heart rate, skin conductivity, eye movements, and brain activity, could be monitored during the use of video games. In turn, these measures could provide useful information about closed-loop adaptation, thorough documentation of training performance and advancement, and real-time feedback [31]. Among video games, several studies have specifically focused on exergames, video games with a focus on physical training [32], which could serve as a motivating factor to encourage consistent physical activity [33]. Exergaming is the term used to describe technology-driven physical activities, like playing video games, that necessitate physical activity or exercise from the players. The user of these games must use their entire body to engage in interactive physical activities such as group fitness exercises, virtual sports, and other similar activities. Exergaming is a concept that transforms a passion for gaming, once thought to be a sedentary activity, into a potentially healthier and more active pastime.

While the use of video games has been reported to be linked to psychopathological risks, such as in the case of Internet gaming disorder (IGD), a role has also been reported in improving cognitive abilities and, in some cases, alleviating symptoms related to depression or other mental conditions [34]. A growing number of studies in the literature are thus stressing the potential benefits of video games when correctly employed in different populations [34].

Working from these premises, this narrative review’s objective is to explore and summarize the body of existing literature on the role of video games in improving cognitive performance, activities in daily life, and symptoms of depression in older individuals with different levels of cognitive impairment. To our knowledge, this is the first review to combine studies focused on the therapeutic use of video games in older adults, with and without mild cognitive impairments or dementia, to improve cognitive functions, psychiatric symptoms, and for the prevention of falls.

## 2. Materials and Methods

Using the electronic databases PubMed, Web of Science, and Scopus in succession, a literature search was carried out between December 2023 and April 2024. Without any filters, limitations, or restrictions, the following search string was used to locate all potentially qualifying records:

((Video games) AND ((elderly) OR (older people) OR (aged) OR (dementia) OR (fall prevention) OR (psychiatric rehabilitation) OR (cognitive impairment) OR (cognitive functions) OR (cognition) OR ((psychiatric disorders) AND ((elderly) OR(older people) OR (aged)))))

Discussions were used to settle eventual disputes among the reviewers over which articles to include. There was a good level of consensus among the reviewers. After a preliminary search and the exclusion of duplicates of possible identified articles, all articles selected for the final evaluation can be found on PubMed.

### 2.1. Inclusion Criteria

- Original research, editorials, and case reports were accepted
- All research examining how video game-based intervention affected a clinically meaningful and health-promoting health outcome was included
- The included studies used objective and reproducible measures to evaluate the results of the intervention, as well as neurocognitive and neuropsychological tests
- The included studies had their full text available in English

### 2.2. Exclusion Criteria

- Reviews or meta-analyses were not accepted
- If the suggested intervention did not fit the standard description of a video game, the study was excluded
- Research that omitted disclosure of how the intervention affected the participants’ cognitive performance was excluded
- Studies that did not use objective and reproducible measures to evaluate intervention outcomes were excluded
- Articles not available in English were excluded

## 3. Results

### 3.1. Video Games to Enhance Cognitive Functions in Older Subjects

It is well-known that some cognitive deficits can be prevented and rehabilitated with the help of cognitive therapies, especially cognitive training. Video games have been recently recognized as a possible resource to be used in cognitive training, thanks to technological advancements. This method makes cognitive work more enjoyable and engaging, which boosts participant motivation and lowers abandonment rates. It also has the added benefit of being more widely available [28,29]. One of the main ideas in the field of cognitive training is the concept of transfer, commonly recognized as the extent to which a learned skill can be demonstrated in a different setting [35]. Near transfer is recognized as an improvement within the same cognitive domain that was trained in, assessed with distinct stimuli, and necessitating a different response than the training task. On the other hand, far transfer is used to describe the development of skills in cognitive domains other than the one being trained in [36]. Executive control abilities, one of the main aspects of executive functioning, are particularly vulnerable to deterioration with aging because of their significant functional reliance on the prefrontal brain and basal ganglia [37,38,39]. It can be assumed that training promoting cognitive flexibility, as in the capacity to modify one’s reactions to the demands of the present environment, would significantly improve the quality of life and daily functioning of older people [40,41]. Within this framework, executive functioning training is acknowledged as one of the domains with the highest potential for fostering flexibility, featuring three main areas: inhibition (as in the repression of ideas or acts, typically in favor of different ideas or acts), updating (as in monitoring incoming information for its relevance and modifying working memory store content if necessary), and shifting (as in switching attention and actions between relevant tasks). However, to date, the available literature about the results of cognitive training in transferring skills across various cognitive abilities in adults is controversial [42]. One of the first studies aiming at evaluating the possibility of training executive functions in an older population, using strategy video games, was conducted by Goldstein et al. (1997), who investigated the effect of the commercial video game Super Tetris in improving cognitive skills in 22 older subjects. The experimental group (n = 10, mean age: 76.5 ± 3.8), who played the game for 5 h a week for 5 weeks, reported a noteworthy amelioration in reaction times compared to the controls (n = 12, mean age: 78.7 ± 6.4) and in cognitive adaptability compared to the baseline [43].

Another study in support of this hypothesis was conducted by Basak et al. (2008). Their research aimed at evaluating the possibility of training executive functions in older adults using a real-time strategy video game called Rise of Nations, combining the intricacy of turn-based strategy games with the pace of real-time gaming. Participants who underwent training, thus playing a real-time strategy video game for a total of 23.5 h (n = 20, mean age 69.10 ± 6.06), showed significant improvement both in game performance and in mental rotation, task switching, working memory, and visual short-term memory compared to the control group (n = 19, mean age: 70.05 ± 4.94). Moreover, enhancements in task-switching were connected with individual variations in gaming performance changes. Results suggested that playing real-time strategy games may have modest but positive effects on executive control functions, associated with frontal lobe’s role in high-level cognitive processes. Given the impact of aging on frontal lobes, especially on the prefrontal cortex, these findings suggest that real-time strategy game training may improve executive control in older individuals, both collectively and individually [44].

Similar results came from a later study conducted by Rosenberg and colleagues, during which 19 older subjects (mean age: 78.7 ± 8.7) residing in community housing underwent weekly sessions of 35 min in length playing Nintendo’s Wii Sports games. After 12 weeks of treatment, the results showed a significant improvement not only in the cognitive performance, but also in the depressive symptoms and overall mental health quality of the subjects. The authors investigated the potential of exergames using the Nintendo Wii Sports platform in 19 older adults (F = 13, M = 6, mean age: 78.7 ± 8.7) with subsyndromal depression symptomatology. Participants were asked to play exergames for a minimum of 30 min, thrice a week, for 12 weeks and were assessed with the Quick Inventory of Depressive Symptoms (Clinician Rated Version) (QIDS), Medical Outcome Study (Short Form) (MOS SF-36), and the Repeatable Battery for Assessment of Neurocognitive Status (RBANS). The outcomes demonstrated a noteworthy amelioration in depressive symptomatology, in cognitive function, and in the overall quality of life connected to mental health [45].

Brem et al. aimed to investigate whether a video game specifically developed for cognitive and cerebral activation could either stop cognitive function from declining or enhance it in hospitalized older adults. For this purpose, the authors compared 16 patients (mean age 66.1 ± 9.3) undergoing daily sessions of video gaming of 30 min in length with 16 control patients (mean age: 69.9 ± 13.9). Results showed that the playing group significantly improved their working memory and fluid intelligence, confirming the positive effects of playing video games on preventing the loss of cognitive function in hospitalized older patients [46].

A pilot study carried out by Gamberini and colleagues addressed the role of video games, and specifically, the Nintendo Wii console, in improving the neuropsychological functions of older people. Participants were tested for memory, attention, and motor control. After a week of gaming, individuals were asked to complete a series of neuropsychological tests assessing selective attention, short-term digit memory, working memory, and visual motor skills. Results showed that sustained and selective attention in the test were correlated and representative of the sum of the average time employed to complete difficult tasks while gaming on the Wii. Furthermore, the authors’ aim was to make neuropsychological tests more attractive to older people by exploiting Nintendo Wii-specific features. Indeed, given its affordability, portability, and small dimensions, the Nintendo Wii could easily be employed directly at home, with the patient being in contact with the rest of the family [47].

Similarly, Peretz et al. (2011) documented how older volunteers (n = 121, mean age: 68 ± 7) that played computer games for three subsequent months improved in focused and sustained attention, memory recognition, and mental flexibility [48]. Maillot et al. (2012) [37] evaluated the potential of 24 one-hour sessions of training with physical activity-based video games (Wii Sports, Wii Fit, and Mario and Sonic Olympics games) to improve cognitive functions in 30 older adults. Results highlighted how subjects who underwent the training (n = 15, mean age: 73.47 ± 4.10) showed significant improvements over the control group in cognitive measures like executive control and processing speed; however, no improvement was detected regarding visuospatial measurements (n = 15, mean age: 73.47 ± 3.00) [49].

A later study carried out by van Muijden et al. (2021) aimed at investigating whether playing cognitive training video games online may enhance cognitive control in older adults. Over a seven-week period, 72 adults aged 60 to 77, either playing cognitive games (n = 53, mean age: 67.8 ± 3.8) or answering documentary quizzes (n = 19, mean age: 67.2 ± 3.4), were evaluated for various cognitive flexibility measures. Results reported that subjects playing video games showed a greater improvement in response inhibition and inductive reasoning than the group that responded to the documentary-style questions. However, measures of other cognitive control aspects did not significantly differ, thus offering only modest support for the hypothesis of the potential of video game training in improving cognitive control in healthy older individuals [36].

Conversely, opposite results have emerged from a recent study conducted by Buitenweg and colleagues (2017). In this study, the authors aimed to examine the effects of an adaptive cognitive flexibility training program that is commercially available on self-perceived cognitive and executive performance, quality of life, depression, and anxiety among 158 participants aged 60 to 80. Eligible participants were divided into a frequent (n = 64, mean age: 67.8 ± 5.0) or infrequent (n = 36, mean age: 67.9 ± 5.4) switching experimental group, or to the active control group (n = 58, mean age: 67.6 ± 5.1) and committed to a 12-week computerized training program. Individuals who had used training games in the past, consumed drugs, had severe vision impairment, were colorblind, had a history of stroke or TIA, or attained a score below 26 on the Telephone Interview for Cognitive Status were excluded. During and after the training period, assessments of the effects on reasoning, verbal long-term memory, planning, processing and psychomotor pace, fluency in speech, and cognitive performance were performed four times. Results demonstrated a notable improvement across the entire sample, not resulting in the predicted near- and far-term transfer ascribable exclusively to the training [42]. Belchior et al. (2019) examined the cognitive and daily functioning of older persons following a 60-session video game-based home intervention. The authors compared three groups: the first playing a video game called Crazy Taxi (n = 17), the second utilizing PositScience InSight, a computerized training program that focuses on visual attention and processing speed (n = 19), and the last receiving no treatment (n = 18). Individuals were assessed for cognitive and everyday functioning prior to and post-training, both immediately after the end of the training and three months later. Outcomes showed that the participants in the video game group benefited less from the intervention in measures of attention and processing speed, but other small benefits were detectable in the video games group, such as a greater improvement in depression symptoms [50].

Another work analyzed the performance of older adults in reaction time and rotation rate, two elements of mental rotation exercise, across two groups with the same previously described interventions (Crazy Taxi and PositScience InSight). Three different groups were formed: an experimental group (n = 17) playing Crazy Taxi, an active control group (n = 19) training with tasks involving fast visual array scan and pattern identification, quick perceptive comparisons, visuospatial working memory, visual discrimination, and selective and divided attention and processing speed, and a passive control group (n = 18). Mental rotation tests conducted at a three-month follow-up demonstrated better performances in the interventional groups, suggesting that both interventions had visuospatial benefits. Other such aspects that could be improved by video games include attentional area of view and rate of visual comparison effectiveness [51].

A study carried out by Ramnath et al. (2021) aimed to examine differences in functional capacity and cognitive performance between traditional multimodal fitness activities and active video gaming treatments in older persons with memory complaints who were recruited from retirement homes. Participants (n = 45, mean age: 72 ± 5) were divided into two groups: the interactive video gaming (IVG) group (n = 23, mean age: 70.8 ± 4.52) and the conventional multimodal (CM) group (n = 22, mean age: 74.14 ± 5.8). The IVG group participated in two one-hour interactive gaming sessions using the X-Box Kinect Sports video game, which included football, ping pong, beach volleyball, boxing, running and jumping, and ten-pin bowling. The CM group engaged in team-based, low-impact, traditional multimodal, supervised exercise programs twice a week for 1 h. Standing and sitting activities were included throughout the sessions, which were led and overseen by an exercise physiologist. The overall training lasted three months. Every clinical measure was completed both before and after the interventions. The following pre-post assessments were used: the Modified Stroop task, the Mini-Mental State Examination, the N-back task, dynamic balance, functional reach, and a six-minute walk. The study’s findings showed that the IVG group significantly outperformed the CM group in the Stroop task and for the average reaction time of correctly colored words. Functional ability, which included the 6-min walk, dynamic balance, timed up-and-go, and functional reach, also showed a considerable improvement. These results suggested a greater effectiveness of interactive video games compared to traditional multimodal exercise in enhancing functional ability and cognitive performance in older persons with memory problems [52].

Notwithstanding the increasing number of articles demonstrating the advantages of using video games in improving the cognitive function of older subjects, many studies have found no differences compared to classic interventions. For instance, Bacha and colleagues (2018) discovered no noteworthy variations in the improvement of cognitive functions measured with the Montreal Cognitive Assessment (MoCA) between subjects (n = 46, mean age: 69.3 ± 5.34) who partook in 14 one-hour long training sessions, twice a week, with Kinect Adventures Games (n = 23) and those who participated in conventional physiotherapy (n = 23) [53]. Similarly, Chuang et al., (2015) compared 26 sedentary older females that underwent either training sessions with the video game Dance Dance Revolution (n = 7, mean age: 69.43 ± 3.82), brisk walking (n = 11, mean age: 67.01 ± 1.67), or no physical activity (n = 8, mean age: 68.25 ± 3.96). While an improvement in inhibitory control was reported compared to the control group, the results did not reveal any noteworthy variations between the brisk walking group and subjects who played the dance video game [54]. Guimaraes et al. (2018) compared the effects of video game physical activity programs (using the Xbox 360 Kinect console) and aerobic exercise on cognitive performance. In the study, a total of 27 older adults were enrolled, with 13 belonging to the experimental group (F = 10, M = 3, mean age: 60.0 ± 4.0) and 14 to the aerobic control group (F = 6, M = 8, mean age: 60.7 ± 3.6). The training lasted 12 weeks and included a session three times a week. Results showed a similar improvement in the cognitive functions of both the experimental and the control group, suggesting the use of physically active video games as a substitute for classical physical exercise [55].

Table 1 displays a synopsis of the studies described.

### 3.2. Video Games in Mild Cognitive Impairment and Dementia

Thanks to the growing interest in employing interactive video games in neuro-rehabilitation as well as in improving both cognition and physical health in older subjects, some authors have hypothesized new strategies to develop novel exergame-based training concepts. In particular, Manser and colleagues suggested a strategy based on an existing methodological framework: the Multidisciplinary Iterative Design of Exergames (MIDE). The authors showed that the development of exergames was facilitated when based on a methodical, incremental, and evidence-based approach, which may allow highlighting of the essential specifications for both the training and exergame components. Consequently, it is anticipated that this will improve the applicability and acceptance of exergame interventions in “real life” situations [56]. In 2008, an investigation was carried out at the University of Porto examining the impact of video games on cognitive abilities, self-perception, and overall quality of life among older individuals. Participants were divided into three groups: an experimental group playing video games (n = 15), an active control group participating in relaxation sessions (n = 17), and a passive control group receiving no experimental treatment (n = 11). All participants were assessed with the Cognitive Su-scale of Alzheimer’s Disease Assessment Scale (ADAS-Cog), the clinical self-concept inventory (ICAC), and the World Health Organization Quality of Life Questionnaire (WHOQOL), both before the intervention and after eight weeks. Results highlighted how, in the experimental group, participants displayed a significant reduction in cognitive decline from before to after the intervention as measured by ADAS-Cog, suggesting that engaging in video games may enhance cognitive functioning as well as sustain self-perception and quality of life in older people. Moreover, the study stressed a positive correlation between self-concept and cognitive benefits, emphasizing the importance of maintaining a positive self-image in achieving cognitive improvements [57].

On the other hand, Yamaguchi et al. (2011) aimed to indirectly improve cognitive function in 18 older people residing in a nursing home, of which 9 had moderate dementia, 1 had Parkinson’s disease, 1 had vascular dementia, and 7 had Alzheimer’s, through session play of sports video games. All participants were assessed with the Revised Hasegawa’s Dementia Scale (HDS-R), the Kohs block design test, and the multidimensional observational scale for older subjects (MOSES). The outcomes demonstrated a noteworthy enhancement across all metrics [58].

In 2014, Hughes and colleagues carried out an investigation to assess whether interactive Wii video games can help older persons with MCI improve their cognitive abilities. The authors enrolled 20 subjects (M = 6, F = 14, mean age: 77.4 ± 5.8 years) who underwent cognitive evaluation by the Computerized Assessment of Mild Cognitive Impairment (CAMCI), the Cognitive Self-Report Questionnaire-25 (CSRQ-25), and Timed Instrumental Activities of Daily Living (TIADL). Outcomes showed that Wii interactive video games were feasible for MCI patients, with bowling being enjoyed by most of participants and showing good effects for stimulation on the cerebral, social, and physical levels [59]. Furthermore, a work was carried out across 22 MCI subjects administered Xbox 360 Kinect cognitive games training, with 22 individuals in the control group. Patients were assessed for cognitive functions through the MMSE and MoCA, and for executive functions using the TMT. The authors concluded that Xbox 360 Kinect video games had positive outcomes for MCI patients following both short- and long-term interventions, and that they could represent potential therapeutic candidates for MCI [60].

Regarding activities of daily life (ADL), a study conducted in Japan by Jahouh et al. (2021) investigated the effects of video games on enhancing fundamental and instrumental ADL performance. The authors investigated whether online games could be correlated with the degree of cognitive impairment and mood in elderly persons living in institutions. During the study, 40 individuals (M = 18, F = 22, mean age: 85.05 ± 8.63 years) administered a video game training regime were compared to a control group (n = 40; M = 17, F = 23; mean age: 83.25 ± 8.78) not involved in gaming activities. The video game employed was the Nintendo Wii Fit and, during sessions, subjects exercised several cognitive functions, including memory and attention, as well as their muscle tone, which kept them balanced during the various tasks. Participants were examined through the Lobo’s Mini-Cognitive Examination (MCE), the Activities of Daily Living test, the Dementia Apathy Interview and Rating (DAIR), and the Goldberg Anxiety and Depression Scale (EADG). Results highlighted how the experimental group improved in terms of cognitive state, levels of ADLs, and overall psychological status. In particular, a decrease in degrees of melancholy, anxiety, and apathy was observed, along with an improvement in memory, focus, and ability to perform fundamental and instrumental ADLs [61].

Another study focused on the assessment of the viability and impact of interactive video games (Xavix Hot Plus) on mental health, mood, and quality of life among individuals with MCI who live in communities. Two groups were formed: one comprising those receiving the intervention (n = 8, M = 3, F = 5, mean age: 79.75 ± 4.86 years) and another by controls (n = 8, M = 3, F = 5, mean age: 77.75 ± 6.74 years). Individuals were assessed for cognitive functions, physical functions, mood status, and standard of living, respectively, by the Short Portable Mental Status Questionnaire (SPMSQ), the Instrumental Activities of Daily Living Scale (IADL), the senior functional test (SFT), the timed up and go test (TUG), the 6-min walk test (6MWT), the timed unipedal stance test (UPST), the Geriatric Depression Scale short form (GDS-SF), the five domains of the Euroqol Health visual analogue scale VAS (EQ5D-VAS), and the five domains of the Euroqol Health utility score (EQ-5D-Utility). Both groups presented increased scores in cognitive functions, and the game showed good levels of feasibility and safeness among people with MCI [62].

A study by Sato et al. (2023) investigated the effects of dance-based video games on 10 subjects with MCI and 11 individuals with average mental abilities. The game was called Step Mania 3.9. The cognitive functions of the participants were assessed using the MMSE, the Japanese version of the MoCA, and the TMT. In addition, prefrontal cortex activity was analyzed. A general assessment was performed before and after the dance video game intervention. In the MCI group, a significant improvement in the Japanese version of the MoCA and in the TMT was registered. Moreover, an increase in dorsolateral prefrontal cortex activity was highlighted in the MCI group after dance video games [63].

Table 2 displays a synopsis of the studies described.

### 3.3. Video Games for Fall Prevention in the Older Subjects

One of the biggest concerns for worldwide public health is falls among older persons, which frequently result in diminished mobility, a worse standard of living, and an increase in mortality and morbidity [64,65]. Since the loss of balance when walking or standing is one of the main causes of falls in older subjects, methods aimed at enhancing standing and walking ability are most likely to decrease the risk of falls [66,67]. Exergame intervention has shown significant promise in improving balance control, being recently tested as a unique rehabilitative technique for individuals with cognitive-motor deficits [19].

In particular, some researchers have focused on the use of home-based step training in older people with video game technology for improving stepping motion and reducing falls. De Bruin et al. (2011) examined how a training program which utilized computer dancing games worked (n = 11; mean age: 86.8 ± 8.1) in comparison to a traditional physical intervention (n = 17; mean age: 85.2 ± 5.5). The experimental group exhibited a higher decrease in fear of falling and a noteworthy improvement in dual walking tasks [68]. During the same year, William et al. (2011) explored the possible benefits of Nintendo’s Wii Fit activities on improving balance in older adults. The study involved 22 subjects (mean age: 83.86 ± 5.47) and lasted 4 weeks, in which participants underwent 20 min training sessions with Wii Fit. Results showed a significant improvement in balance scores after 12 weeks of training [69]. Similarly, Picchierri and colleagues investigated the effect of training with dance video games, comparing it to traditional training. For the purpose of the study, the authors recruited a sample of 31 older subjects (mean age: 86.2  ±  4.6) who were then divided into an experimental group that, in addition to a traditional training consisting of 5 min of warm up, 25 min of resistance training, and 10 min of balance exercise, also trained twice a week with the dance video game (n = 15), and a control group (n = 16) that performed only strength and balance exercises. Results showed a significant improvement in step time and fast walking performance under dual task conditions in favor of the experimental group, suggesting a greater efficiency of the experimental training program [70]. Schoene et al. (2013) enrolled 15 older subjects (mean age: 77.5 ± 4.5) who were asked to play a computer game where they were required to adhere to the directions on a monitor by stepping on a step pad (e.g., Dance Dance Revolution (DDR); Konami) and 17 controls (mean age: 78.4 ± 4.5), who continued their usual activities of daily life. The game consisted of stepping repeatedly in all directions at various speeds and challenged balance, coordination, reaction time, and attention. Participants were assessed for cognitive functions through the choice stepping reaction time (CSRT) and the Physiological Profile Assessment (PPA) at baseline and at a follow-up of eight weeks. The findings indicated that older adults without significant cognitive or physical impairments can safely perform step pad training at home to enhance physical and cognitive characteristics and lower their risk of falling [71]. Those results where later confirmed by a study from Nicholson et al. (2015) that aimed to evaluate unattended Nintendo Wii Fit balancing training’s efficacy and safety in older subjects. The overall sample consisted of 41 subjects recruited from retirement villages. Those subjects were then divided into an experimental group (n = 19, mean age: 75 ± 6) that underwent a program of 30 min of unattended Wii balance gaming thrice a week, and a control group (n = 22, mean age: 74 ± 5) that performed a traditional training program. Results highlighted how the experimental group had a significantly greater improvement in timed up-and-go, left single-leg balance, both lateral reach, and gait speed compared with the controls [72].

Despite these promising results, other research has failed to confirm the advantages of using video game-based interventions as compared to standard ones. In particular, Kliem et al. (2010) compared a traditional training program to a training program which used the Nintendo Wii Fit TM Balance Board. Results showed that the traditional training led to a significantly greater improvement in two tests (SEBT and ball-handling), while the experimental group improved significantly only in the ski slalom, suggesting that although the video game platform could represent a suitable substitution, traditional training still maintains greater efficiency [73].

Results of the studies discussed above are summarized in Table 3.

### 3.4. Video Games for Psychiatric Disorders in Older Subjects

Psychiatric symptoms, and particularly subsyndromal depression, are highly prevalent in the older population and are linked to significant suffering, functional impairment, higher mortality, and increased use of expensive medical services [74,75]. Numerous randomized controlled trials have investigated the impact of exercise therapies on depressive symptoms, and results are encouraging when an exercise regimen is followed [76,77]. Unfortunately, when the training program is conducted at home, these interventions frequently lack safety and are beset by high rates of non-adherence, with a drop-out rate of roughly 50% within three to six months. Exergames, enjoyable video games that use physical input devices to mix game play with significant physical training, are becoming increasingly relevant and popular as a substitution for traditional programs.

Interestingly, many researchers have suggested a possible role of exergames in the control of depressive symptoms in older subjects. In a recent review, which analyzed the literature about the use of exergames for improving depressive symptoms in older people (including both studies that considered exergames as proper games and those that considered them simply technology-driven exercise) the authors highlighted, on the basis of 17 studies, a general improvement in depressive symptomatology, in particular among subjects with previous depressive symptoms [78].

Li et al. (2016) investigated whether the playfulness inherent of exergames could be useful in the treatment of depressive symptomatology in 49 older adults (F = 29, M = 20, mean age: 71.12 ± 8.67) diagnosed with subthreshold depression. The authors compared two experimental groups: the first, named high playfulness (n = 25, mean age: 71.20 ± 8.86), involved the use of Wii Sports games, and the second, labeled low playfulness, involved the use of Wii Fit training (n = 24, mean age: 71.04 ± 8.65). Results demonstrated a reduction in depressive symptoms in both groups, as well as greater positive emotions and self-efficacy. Interestingly, playfulness was found to have an effect only on positive emotions, and not the overall depressive symptoms [79].

Yang et al. (2017) aimed to investigate the effect of a virtual reality exergame on depressive symptomatology in older subjects with mild depressive symptoms. The study involved a total of 15 community-dwelling subjects (mean age: 70.0 ± 5.94) who voluntarily selected three 10 min long exercise programs, between four activities. The group trained for 45 min, thrice per week, for a total of 12 weeks. Outcomes highlighted a significant improvement in both depressive symptoms and internal stress scores [80].

Rodrigues et al. (2018) investigated the potential of pop dance exergaming to improve depressive symptoms and reduce fear of falling and falling risk. The study recruited a total of 47 community-dwelling older women, divided into an experimental group (n = 22) and a control group (n = 25), both including fallers and non-fallers. The experimental group played dancing video games (Dance Central game for Xbox 360) thrice a week for a total of 12 weeks. Results showed a significant improvement in the depressive symptomatology in the fallers of the experimental group [81].

A recent study conducted by Heinbach and colleagues (2021), evaluated the impact of exergames on depressive and negative symptoms in older people with serious mental illness. The study was conducted on a sample of 52 older people (M = 32, F = 20, mean age: 59.2 ± 5.3). Participants underwent 50 min exergames sessions thrice a week for a total of 10 weeks. At enrollment and at five and ten weeks after the start of the study, patients were assessed using the Patient Reported Outcome Measurement Information System (PROMIS) and the Scale for the Assessment of Negative Symptoms (SANS). At the end of the 10 weeks of observation, participants presented statistically significant reductions in depressive and negative symptoms, suggesting the possible effectiveness of exergames in treating these problems in older people diagnosed with serious mental illness [82].

In the same year, de Lima and colleagues evaluated the effects of exergames using the Xbox Kinetic on sleep quality, anxiety, and functional capacity in a sample of 29 older people. Candidates were divided in two groups: an experimental group (n = 15, mean age: 67.2 ± 4.4) and a control group (n = 14, mean age: 68.0 ± 6.1). The experimental group underwent exergame sessions lasting 60 min thrice a week for six weeks using the Xbox Kinetic platform; the control group did not perform any exercises. Significant improvements were noted in the experimental group at the conclusion of the trial, not only in sleep quality and anxiety, but also in aerobic endurance, agility/balance, and lower limb strength [83]. A study by Otero et al. (2021) investigated the effects of interactive multimedia online active video games to promote active aging and healthy lifestyles habits as well as to prevent depression. The authors enrolled 25 participants (M = 9, F = 16, mean age: 54.9 years) who received a three-pronged multicomponent therapy: cognitive stimulation, healthy lifestyle modification, and depression prevention. The primary results of the intervention, which was carried out through an interactive online multimedia game (graphic adventure style) with a companion smartphone app, showed improvements in overall well-being, bodily processes, social interactions, and mental health. It was shown that video games are effective and feasible in promoting active aging [84].

Table 4 displays a synopsis of the studies described.

## 4. Discussion

Globally, the above reviewed studies have revealed that older subjects trained with video games showed a significant improvement in cognitive functions, depressive symptoms [45,79,80,81,82,83,84], sleep quality, and anxiety [83]. Other studies have reported that older people trained with video game sessions experienced a reduction in depression and anxiety levels, improved memory and attention, and consequent favorable effects on daily life activities [50,51]. On the basis of these data, video games appear to be an intriguing and useful tool for the improvement of cognitive function, depression symptoms, and positive emotions in the older population. However, not all of the studies have replicated these results [55]. It should be noted that studies in this field suffer from great heterogeneity in protocols and kinds of video games used, which undoubtedly impact on the replicability of the results. In addition, other studies in different populations have pointed out that the combination of the specific individual approach to video games, including the grade of interest and involvement in the activity, and the specific kind of video game itself may lead to differences and sometime opposite effects on neurobiological and psychological outcomes [32].

Moreover, some authors have stressed possible different effects of video games and other kinds of computer training on cognitive function and mental health symptoms. For example, in the study of Belchior et al. (2019), subjects trained with the video game Crazy Taxi showed lower improvement in visual attention and processing speed than subjects that employed a specific computer-based training for these functions. However, Crazy Taxi users reported different patterns of improvement in other areas, such as a greater improvement in depressive symptoms. Despite that, most of the available literature about the use of video games for depressive symptoms in this population has focused on exergames, and given the study of Belchior et al. (2019), more studies should focus also on the effects of other types of video games [50]. Controversial results have emerged regarding the use of exergames for preventing falls, with studies alternatively reporting a greater or lower efficiency of exergames in fall prevention with respect to standard technique [72,73].

Possible limitations of the studies examined include small sample sizes, short study durations, volunteer participants with higher motivation to perform the exergames, drop-out from poorly motivated patients during follow-up, interfering activities performed during the study period, and lack of a control group [50,79,83]. Furthermore, the narrative nature of our review may have led to selection bias in the articles. Despite the limitations of the available studies, the results suggest that the older population may benefit from video games to improve depressive symptoms, cognitive functions, and quality of life, while also remembering to pay attention to the possible negative effects of video games, including their addictive power [32].

Further research is needed to evaluate possible benefits and issues surrounding the use of video games in the older population, using larger sample sizes and more standardized protocols. The information gained from these studies may also enable the implementation and development of video games with designs specifically tailored for their use in the clinical and rehabilitative field.

## Figures and Tables

**Table 1:** Studies investigating the use of video games for improving cognitive functioning in older people without dementia and MCI.

Authors	Sample	Intervention	Measures	Results
Basak et al. (2008) [44]	Total sample: n = 39; experimental group: n = 20, (mean age: 69.10 ± 6.06); control group: n = 19, (mean age: 70.05 ± 4.94).	Sessions of real-time strategy video game for a total of 23.5 h	Executive control tasks (operation span, task switching, N-back test, visual short-term memory, Raven’s Advanced Matrices, stopping task) and visuo-spatial attentional tasks (functional field of view, attentional blink, enumeration, mental rotation)	Modest but positive effects of playing real-time strategy games on executive control functions
Van Muijden et al. (2012) [36]	Total sample: n = 72 (F = 32; M = 40); experimental group: n = 53 (F = 28; M = 25, mean age: 67.8 ± 3.8); control group: n = 19 (F = 4, M = 15, mean age: 67.2 ± 3.4)	Experimental group: playing cognitive games; Control group: answering documentary quizzes	MMSE, Stroop Color-Word Test, Stop-Signal Test, counting span, Mental Counters task, Useful Field of View Test, Raven-SPM, Global-Local Switching Test, Smiling Faces Switching Test, Test of Attentional Performance	Greater improvement in response inhibition and inductive reasoning in subjects playing video games. Measures of other cognitive control aspects did not significantly differ.
Buitenweg et al. (2017) [42]	Total sample: n = 158; frequent switching: n = 64 (F = 41; M = 23; mean age: 67.8 ± 5.0); infrequent switching: n = 36 (F = 23; M = 13; mean age: 67.9 ± 5.4); controls: n = 58 (F = 31; M = 27; mean age: 67.6 ± 5.1)	Cognitive training: nine games in three domains (reasoning, working memory, and attention); FS: ten games of 3 min each; IS: three games of 10 min each Mock training: four games played in sessions of three games of 10 min each	Stop-signal task, SSRT, D-KEFS TMT, TMT-B/A, drag-and-drop task, drag-to-grid task, click task, DSC, Neurotask BV, ToL, Raven-SPM, Shipley Institute of living scale-2, Corsi block tapping task, PASAT, Operation Span, RAVLT, LNS, CIS-F, HADS-D	Significant improvement in the overall sample
Belchior et al. (2019) [50]	Total sample: n = 54, mean age 63 ± 6; experimental group: n = 17; computer training: n = 19; control group: n = 18	Experimental group: 3 months of home practice for 60 h of Crazy Taxi Computer training: 3 months of home practice for 60 h of Crazy Taxi	Primary outcomes: speed, divided attention, selective attention, same-different. Secondary outcomes: visual attention influenced by video games, visual spatial skills, everyday function, subjective functioning	Improvement in both training groups. In computer training, greater improvement in visual attention and processing speed; other benefits in Crazy Taxi group
Goldstein et al. (1997) [43]	Total sample: n = 22; experimental group: n = 10, mean age: 76.5 ± 3.8; control group: n = 12, mean age: 78.7 ± 6.4	Super Tetris for 5 h a week for 5 weeks	Sternberg Test, Stroop Color Word Test	Significant improvement in experimental group in reaction times. Significant improvement in cognitive adaptability compared to the baseline.
Basak et al. (2008) [44]	Total sample: n = 39; experimental group: n = 20, mean age 69.10 ± 6.06; control group: n = 19, mean age: 70.05 ± 4.94.	Sessions of real-time strategy video game for a total of 23.5 h	Executive control tasks (operation span, task switching, N-back test, visual short-term memory, Raven’s Advanced Matrices, stopping task) and visuo-spatial attentional tasks (functional field of view, attentional blink, enumeration, mental rotation)	Modest but positive effects of playing real-time strategy games on executive control functions
Rosemberg et al. (2010) [45]	Total sample: n = 19, mean age: 78.7 ± 8.7	12 weeks of 35 minute-long weekly sessions of Nintendo’s Wii Sports games	QIDS; BAI; RBANS	Notable improvements in depression symptoms, cognitive function, and general mental wellness.
Brem et al. (2010) [46]	Experimental group: n = 16 (F = 10, M = 6, mean age 66.1 ± 9.3); control group: n = 16 (F = 10, M = 6, mean age: 69.9 ± 13.9)	30 minute-long daily sessions of video gaming	Kurztest fur Allgemeine Intelligenz, 12-item Short Form health survey, Neuroticism-extroversion-openness five factor inventory, patient reported outcom scale	Notable enhancements in working memory and fluid intelligence in the playing group.
Gamberini et al. (2010) [47]	Older adults	Nintendo Wii video games and standardized paper and pencil (PP) neuropsychological tests after 1 week of video gaming sessions	Cognition, memory, and attention were assessed	A positive correlation was found between video games and PP neuropsychological tests, except the digit span.
Peretz et al. (2011) [48]	Total sample: n = 121, F = 77, M = 44, mean age: 68 ± 7	24 sessions of 20–30 min of computer games, thrice a week, for 3 months	NexAde cognitive test battery, Geriatric Depression Scale	Enhanced cognitive flexibility, memory recognition, and focused and sustained attention.
Maillot et al. (2012) [49]	Total sample: n = 30; experimental group: n = 15, mean age: 73.47 ± 4.10; control group: n = 15, mean age: 73.47 ± 3.00	24 one-hour training sessions with Wii Sports, Wii Fit, and and Mario and Sonic Olympics games	Trail making test, Stroop color word interference test, Letter sets test, matrix reasoning test, digit symbol substitution test, spatial span test, directional heading test, mental rotation test, cancellation test, number comparison test, reaction time test, plate tapping test	Subjects who underwent the training showed a significant improvement in cognitive measures such as executive control and processing speed, but not in visuospatial measures.
Chuang et al. (2015) [54]	Total sample: F = 26; DDR group: F = 7, mean age: 69.43 ± 3.82; BW group: F = 11, mean age: 67.01 ± 1.67; control group: F = 8, mean age: 68.25 ± 3.96	DDR group: three sessions of 30 min exercise with DDR BW group: three sessions of 30 min of BW	Flanker task	Improved inhibitory control in DDR and BW groups. No significant differences between DDR and BW groups.
Bacha et al. (2018) [53]	Total sample: n = 46, mean age: 69.3 ± 5.34; experimental group: n = 23; control group: n = 23	14 one-hour long training sessions, twice a week, with Kinect Adventures Games	MoCA	No differences in MoCA test improvements between groups.
Guimaraes et al. (2018) [55]	Total sample: n = 27; experimental group: n = 13 (F = 10, M = 3, mean age: 60.0 ± 4.0); control group: n = 14 (F = 6, M = 8, mean age: 60.7 ± 3.6)	A session three times a week for 12 weeks	CogState battery	Similar improvement in the cognitive functions of both groups.
Taylor et al. (2021) [51]	Total sample: n = 54, mean age: 73 ± 6; experimental group: n = 17; active control group: n = 17; passive control group: n = 18	Experimental control: Crazy Taxi playing sessions Active control: cognitive training		Better performances in mental rotation tests
Ramnath et al. (2021) [52]	Total sample: n = 45, mean age: 72.4 ± 5.37; experimental group: n = 23, mean age: 70.8 ± 4.52; control group: n = 22, mean age: 74.14 ± 5.8	Experimental group: two 1 h long interactive video game sessions with the X-Box Kinect Sports video game. Control group: 1 h long low intensity conventional multimodal supervised exercise sessions, twice a week	Mini-Mental State Examination, N-back Task, Modified Stroop task	Significant improvement in the IVG group compared to the CM group in the Stroop task and for average reaction time to correct color-words.

MMSE: Mini mental state examination; Raven-SPM: Raven’s Standard Progressive Matrices; SSRT: Stop-signal reaction time; D-KEFS TMT: Delis-Kaplan Executive Function System—Trail Making Test; TMT-B/A: Trail Making Test B/A; DSC: Digit Symbol Coding test; ToL: Tower of London; PASAT: Paced Auditory Serial Addition Task; RAVLT: Rey’s Auditory Verbal Learning Test; LNS: Letter Number Sequencing; CIS-F: Checklist Individual Strength, fatigue subscale; HADS-D: Hospital Anxiety Depression Scale, depression subscale; QIDS: Quick Inventory of Depressive Symptoms; BAI: Beck Anxiety Inventory; RBANS: Repeatable Battery for Assessment of Neurocognitive Status; HDS-R: Hasegawa’s dementia scale revised; MOSES: multidimensional observational scale for elderly subjects; DDR: Dance Dance Revolution; BW: brisk walking: MoCA: Montreal Cognitive Assessment; IVG: Interactive Video Gaming; CM: Conventional Multimodal.

**Table 2:** Studies investigating the use of video games in older people with MCI and dementia.

Authors	Sample	Intervention	Measures	Results
Torres et al. (2008) [57]	Total sample: n = 43; experimental group: n = 15; active control group: n = 17; passive control group: n = 11. Mean age = 78.33 ± 8.002.	Experimental group: video game playing sessions; active control group: relaxation sessions; passive control group: no intervention	ADAS-Cog, ICAC, WHOQOL	Significant reduction in cognitive decline from before to after intervention in the experimental group. *Positive correlation between self-concept and cognitive benefits.*
Yamaguchi et al. (2011) [58]	Total sample: n = 18	Sport video games sessions	HDS-R, Kohs block design test, MOSES	Significant improvement across all measures
Jahouh et al. (2021) [61]	Total sample: n = 80; experimental group: n = 40 (M = 18, F = 22, mean age: 85.05 ± 8.63); control group: n = 40 (M = 17, F = 23, mean age: 83.25 ± 8.78)	20 rehabilitation sessions over 8 weeks, made up of different activities with the Nintendo Wii Fit	MCE, Activities of Daily Living test, DAIR, EADG	Reduced depression, anxiety, and apathy levels, and improved memory, attention, and performance of fundamental and instrumental ADL in the experimental group
Hughes et al. (2014) [59]	Total sample: n = 20 (M = 6, F = 14, mean age: 77.4 ± 5.8)	Wii interactive video games sessions	CAMCI, CSRQ-25, TIADL.	Interactive video games are feasible for MCI individuals, and exert good effects on social, mental, and physical stimulation
Amjad et al. (2019) [60]	Total sample: n = 44; experimental group: n = 22; control group: n = 22.	Xbox 360 Kinect cognitive games training	MMSE, MoCA, TMT	The 360 Kinect video game had positive outcomes for MCI patients following both short- and long-term intervention
Lin et al. (2022) [62]	Total sample: n = 16; experimental group: n = 8 (M = 3, F = 5 mean age: 79.75 ± 4.86; control group: n = 8 (M = 3, F = 5, mean age: 77.75 ± 6.74)	Interactive video games (Xavix Hot Plus)	*SPMSQ*; *IADL*; SFT: TUG; 6MWT; UPST; GDS-SF; EQ5D-VAS; EQ-5D-Utility	Increased scores in cognitive functions and good levels of feasibility and safeness among people with MCI.
Sato et al. (2023) [63]	Total sample: n = 21; experimental group: n = 10 (mean age: 77.7 ± 5.1); control group = 11 (mean age: 74.1 ± 4.7)	Dance video games (sessions of Step Mania 3.9)	MMSE, Japanase version of MoCA, TMT.	In the MCI group, a significant improvement in the Japanese version of the MoCA and in the TMT, as well as an increase in dorsolateral prefrontal cortex activity.

WHOQOL: World Health Organization Quality of Life Questionnaire; MCE: Lobo’s Mini-Cognitive Examination; DAIR: Dementia Apathy Interview and Rating; EADG; Goldberg Anxiety and Depression Scale; CAMCI: Computerized Assessment of Mild Cognitive Impairment; CSRQ-25: Cognitive Self-Report Questionnaire-25; TIADL: Timed Instrumental Activities of Daily Living; MCI: mild cognitive impairment; MMSE: Mini Mental State Examination; MoCA: Montreal Cognitive Assessment; TMT: trail making test; SPMSQ: Short Portable Mental Status Questionnaire; IADL: the Instrumental Activities of Daily Living Scale; SFT: senior functional test; TUG: timed up-and-go test; 6MWT: 6-min walk test; UPST: timed unipedal stance test; GDS-SF: Geriatric Depression Scale short form; VAS (EQ5D-VAS): five domains of the Euroqol Health visual analogue scale; EQ-5D-Utility: five domains of the Euroqol Health utility score.

**Table 3:** Studies investigating the use of video games for fall prevention in older people.

Authors	Sample	Intervention	Measures	Results
De Bruin et al. (2011) [68]	Total sample: n = 28; experimental group: n = 11 (mean age: 86.8 ± 8.1); control group: n = 17 (mean age: 85.2 ± 5.5)	Computer dancing games	Gait assessment, ETGUG, fear of falling	Significant improvement in dual tasks of walking and increased decrease in fear of falling in the experimental group
William et al. (2011) [69]	Total sample: n = 22, mean age: 83.86 ± 5.47	20 min training sessions with Nintendo’s Wii Fit activities	BBS	Significant improvement in balance scores after 12 weeks of training
Picchierri et al. (2012) [70]	Total sample: n = 31, mean age: 86.2 ± 4.6; experimental group: n = 15 Control group: n = 16	Training twice a week with a dance video game	PPA, FPA, FES-I, gaze behaviour, gait analysis	Significant improvement in step time and fast walking performance under dual task conditions in the experimental group
Kliem et al. (2010) [73]	Total sample: n = 22 (mean age 47.6 ± 13.1)	Experimental group: training with the Nintendo Wii Fit TM Balance Board Control group: traditional training	SEBT, ball-handling, ski slalom, balance bubble, dynamic balance	Control group improved in SEBT and ball-handling; experimental group improved only in the ski slalom
Schoene et al. (2013) [71]	Total sample: n = 32; experimental group: n = 15, mean age: 77.5 ± 4.5; control group: n = 17, mean age. 78.4 ± 4.5	Dance Dance Revolution, 2–3 sessions per week for 15–20 min over 8 weeks	CSRT, PPA	Experimental group improved their CSRT and PPA scores, their postural sway, and contrast sensitivity
Nicholson et al. (2015) [72]	Total sample: n = 41 (mean age: 74.5 ± 5.4); experimental group: n = 19 (F = 12; M = 7, mean age: 75 ± 6); control group: n = 22 (F = 15; M = 7, mean age: 74 ± 5)	30 min of unsupervised Wii balance gaming three times a week	Timed up-and-go, left single-leg balance, lateral reach, gait speed	Significantly greater improvement in timed up-and-go, left single-leg balance, both lateral reach, and gait speed in the experimental group

CSRT: Choice Stepping Reaction Time; PPA: Physiological Profile Assessment; FPA: Foot placement accuracy; FES-I: Falls Efficacy Scale International; ETGUG: Extended time get-up-and-go; BBS: Berg Balance Scale.

**Table 4:** Studies investigating the use of video games in the treatment of depression in older people.

Authors	Sample	Intervention	Measures	Results
Li et al. (2016) [79]	Total sample: n = 49 (F = 29, M = 20, mean age: 71.12 ± 8.67); high playfulness: n = 25 (F = 14, M = 11, mean age: 71.20 ± 8.86); low playfulness: n = 24 (F = 15, M = 9, mean age: 71.04 ± 8.65)	High playfulness: exergaming sessions with Wii Sports Low playfulness: exergaming sessions with Wii Fit training	PHQ-9, PANAS	Improvement in depressive symptoms, positive emotions, and self-efficacy in both groups. Playfulness affected only positive emotions.
Yang et al. (2017) [80]	Total sample: n = 15 (F = 10, M = 5, mean age: 70.0 ± 5.94)	45 min sessions three times per week for a total of 12 weeks with Wii Fit	GDS	Significant improvement in both depressive symptoms and internal stress scores
Rodrigues et al. (2018) [81]	Total sample: n = 47; experimental group: n = 22 (fallers = 10, mean age: 69.8 ± 4.3; nonfallers = 12, mean age: 68.9 ± 3.3); control group: n = 25 (fallers = 12, mean age: 73.6 ± 5.4; nonfallers = 13, mean age: 68.7 ± 4.8)	30 min training session with Dance Central game for Xbox 360 three times a week for a total of 12 weeks	GDS, FES-I Brazil	Significant improvement in depressive symptomatology in the fallers of the experimental group
Heinbach et al. (2021) [82]	Total sample: n = 52 (F = 20, M = 32, mean age: 59.2 ± 5.3)	50 min exergames sessions three times a week for a total of 10 weeks	PROMIS, SANS	Significant reductions in depressive and negative symptoms.
de Lima et al. (2021) [83]	Total sample: n = 29 (F = 23, M = 6); experimental group: n = 15 (F = 13, M = 2, mean age: 67.2 ± 4.4); control group: n = 14 (F = 10, M = 4, mean age: 68.0 ± 6.1)	60 min session three times per week for 6 weeks using the Xbox Kinect game “Your Shape: Fitness Evolved”	PSQI, STAI	Significant improvement in sleep quality and anxiety in the experimental group
Otero et al. (2021) [84]	Total sample: n = 25 (F = 16, M = 9, mean age: 54.9)	Multicomponent intervention by three components: depression prevention, healthy lifestyle and cognitive stimulation	General health, physical functioning, social functioning, and mental health	Video games are effective and feasible in promoting active aging.

QIDS: Quick Inventory of Depressive Symptoms: Clinician Rated Version; MOS SF-36: Medical Outcome Study, Short Form; RBANS: Repeatable Battery for Assessment of Neurocognitive Status; PHQ-9: Patient Health Questionnaire; PANAS: Positive and Negative Affect Schedule; GSE: general self-efficacy; GDS: Geriatric Depression Scale; FES-I: Fall Efficacy Scale, International; PROMIS: Patient Reported Outcome Measurement Information System; SANS: Scale for the Assessment of Negative Symptoms; PSQI: Pittsburgh Sleep Quality Index; STAI: Spielberger State-Trait Anxiety Inventory.

## Data Availability

All data generated or analyzed during this study are included in this published article.

## References

[1] Petersen R.C., Smith G.E., Waring S.C., Ivnik R.J., Tangalos E.G., Kokmen E. (1999). Mild cognitive impairment: Clinical characterization and outcome. Arch. Neurol..

[2] American Psychiatric Association (2013). Diagnostic and Statistical Manual of Mental Disorders.

[3] Yu J., Lam C.L., Lee T.M. (2017). White matter microstructural abnormalities in amnestic mild cognitive impairment: A meta-analysis of whole-brain and ROI-based studies. Neurosci. Biobehav. Rev..

[4] Petersen R.C., Bennett D. (2005). Mild cognitive impairment: Is it Alzheimer’s disease or not?. J. Alzheimers Dis..

[5] Petersen R.C. (2011). Clinical practice. Mild cognitive impairment. N. Engl. J. Med..

[6] Roberts R.O., Knopman D.S., Mielke M.M., Cha R.H., Pankratz V.S., Christianson T.J., Geda Y.E., Boeve B.F., Ivnik R.J., Tangalos E.G. (2014). Higher risk of progression to dementia in mild cognitive impairment cases who revert to normal. Neurology.

[7] Petersen R.C., Lopez O., Armstrong M.J., Getchius T.S.D., Ganguli M., Gloss D., Gronseth G.S., Marson D., Pringsheim T., Day G.S. (2018). Practice guideline update summary: Mild cognitive impairment: Report of the Guideline Development, Dissemination, and Implementation Subcommittee of the American Academy of Neurology. Neurology.

[8] Brun A., Liu X., Erikson C. (1995). Synapse loss and gliosis in the molecular layer of the cerebral cortex in Alzheimer’s disease and in frontal lobe degeneration. Neurodegeneration.

[9] Miller B.L., Seeley W.W., Mychack P., Rosen H.J., Mena I., Boone K. (2001). Neuroanatomy of the self: Evidence from patients with frontotemporal dementia. Neurology.

[10] Elahi F.M., Miller B.L. (2017). A clinicopathological approach to the diagnosis of dementia. Nat. Rev. Neurol..

[11] Olazaran J., Reisberg B., Clare L., Cruz I., Peña-Casanova J., Del Ser T., Woods B., Beck C., Auer S., Lai C. (2010). Nonpharmacological therapies in Alzheimer’s disease: A systematic review of efficacy. Dement. Geriatr. Cogn. Disord..

[12] Scales K., Zimmerman S., Miller S.J. (2018). Evidence-Based nonpharmacological practices to address behavioral and psychological symptoms of dementia. Gerontologist.

[13] Sikkes S.A.M., Tang Y., Jutten R.J., Wesselman L.M.P., Turkstra L.S., Brodaty H., Clare L., Cassidy-Eagle E., Cox K.L., Chételat G. (2021). Toward a theory-based specification of non-pharmacological treatments in aging and dementia: Focused reviews and methodological recommendations. Alzheimers Dement..

[14] Petersen R.C. (2016). Mild cognitive impairment. CONTINUUM Lifelong Learn. Neurol..

[15] Jessen F., Amariglio R.E., van Boxtel M., Breteler M., Ceccaldi M., Chételat G., Dubois B., Dufouil C., Ellis K.A., van der Flier W.M. (2014). A conceptual framework for research on subjective cognitive decline in preclinical Alzheimer’s disease. Alzheimers Dement..

[16] Aguirre E., Woods R.T., Spector A., Orrell M. (2013). Cognitive stimulation for dementia: A systematic review of the evidence of effectiveness from randomised controlled trials. Ageing Res. Rev..

[17] Keogh J.W., Power N., Wooller L., Lucas P., Whatman C. (2014). Physical and psychosocial function in residential aged-care elders: Effect of Nintendo Wii Sports games. J. Aging Phys. Act..

[18] Fonte C., Smania N., Pedrinolla A., Munari D., Gandolfi M., Picelli A., Varalta V., Benetti M.V., Brugnera A., Federico A. (2019). Comparison between physical and cognitive treatment in patients with MCI and Alzheimer’s disease. Aging.

[19] Chen Y., Zhang Y., Guo Z., Bao D., Zhou J. (2021). Comparison between the effects of exergame intervention and traditional physical training on improving balance and fall prevention in healthy older adults: A systematic review and meta-analysis. J. Neuroeng. Rehabil..

[20] Liao Y.Y., Tseng H.Y., Lin Y.J., Wang C.J., Hsu W.C. (2020). Using virtual reality-based training to improve cognitive function, instrumental activities of daily living and neural efficiency in older adults with mild cognitive impairment. Eur. J. Phys. Rehabil. Med..

[21] Tortora C., Di Crosta A., La Malva P., Prete G., Ceccato I., Mammarella N., Di Domenico A., Palumbo R. (2024). Virtual reality and cognitive rehabilitation for older adults with mild cognitive impairment: A systematic review. Ageing Res. Rev..

[22] Sokolov A.A., Collignon A., Bieler-Aeschlimann M. (2020). Serious video games and virtual reality for prevention and neurorehabilitation of cognitive decline because of aging and neurodegeneration. Curr. Opin. Neurol..

[23] Primack B.A., Carroll M.V., McNamara M., Klem M.L., King B., Rich M., Chan C.W., Nayak S. (2012). Role of video games in improving health-related outcomes: A systematic review. Am. J. Prev. Med..

[24] Cardoso N.d.O., Landenberger T., Argimon I.d.L. (2017). Jogos Eletrônicos como Instrumentos de Intervenção no Declínio Cognitivo—Uma Revisão Sistemática. Rev. Psicol. IMED.

[25] Alves F.d.O.d.S., Oliveira D.C.A., Borges J.d.S. (2020). Um olhar para o uso de tecnologias no cuidado de idosos com deficiência cognitiva em tempos de pandemia. Rev. Sci. Hist..

[26] Entertainment Software Association (2010). Game Player Data.

[27] Ishibashi G.A., Santos G.D., Moreira A.P.B., Verga C.E.R., Silva G.A.D., Ordonez T.N., Moraes L.C., Lessa P.P., Brucki S.M.D., Silva T.B.L.D. (2023). Effects of cognitive interventions with video games on cognition in healthy elderly people: A systematic review. Arq. Neuropsiquiatr..

[28] Dassen F.C.M., Houben K., Van Breukelen G.J.P., Jansen A. (2018). Gamified working memory training in overweight individuals reduces food intake but not body weight. Appetite.

[29] Contreras-Somoza L.M., Irazoki E., Toribio-Guzmán J.M. (2021). Usability and User Experience of Cognitive Intervention Technologies for Elderly People With MCI or Dementia: A Systematic Review. Front. Psychol..

[30] Michalski S.C., Szpak A., Saredakis D., Ross T.J., Billinghurst M., Loetscher T. (2019). Getting your game on: Using virtual reality to improve real table tennis skills. PLoS ONE.

[31] deBettencourt M.T., Cohen J.D., Lee R.F., Norman K.A., Turk-Browne N.B. (2015). Closed-loop training of attention with real-time brain imaging. Nat. Neurosci..

[32] Kooiman B.J., Sheehan D.P. (2015). Interacting with the past, present, and future of exergames: At the beginning of a new life cycle of video games?. Soc. Leis..

[33] Stojan R., Voelcker-Rehage C. (2019). A Systematic Review on the Cognitive Benefits and Neurophysiological Correlates of Exergaming in Healthy Older Adults. J. Clin. Med..

[34] Carpita B., Muti D., Nardi B., Benedetti F., Cappelli. A., Cremone I.M., Carmassi C., Dell’Osso L. (2021). Biochemical Correlates of Video Game Use: From Physiology to Pathology. A Narrative Review. Life.

[35] Buitenweg J.I., Murre J.M., Ridderinkhof K.R. (2012). Brain training in progress: A review of trainability in healthy seniors. Front. Hum. Neurosci..

[36] van Muijden J., Band G.P., Hommel B. (2012). Online games training aging brains: Limited transfer to cognitive control functions. Front. Hum. Neurosci..

[37] Ridderinkhof K.R., Van Den Wildenberg W.P.M., Segalowitz S.J., Carter C.S. (2004). Neurocognitive mechanisms of cognitive control: The role of prefrontal cortex in action selection, response inhibition, performance monitoring, and reward-based learning. Brain Cogn..

[38] Treitz F.H., Heyder K., Daum I. (2007). Differential course of executive control changes during normal aging. Neuropsychology, Development, and Cognition. Sect. B Aging Neuropsychol. Cogn./Aging Neuropsychol. Cogn..

[39] Roberts B.A., Fuhrer R., Marmot M., Richards M. (2011). Does retirement influence cognitive performance? the whitehall II study. J. Epidemiol. Community Health.

[40] Buchler N.G., Hoyer W.J., Cerella J. (2008). Rules and more rules: The effects of multiple tasks, extensive training, and aging on task-switching performance. Mem. Cognit..

[41] Karbach J., Kray J. (2009). How useful is executive control training? Age differences in near and far transfer of task-switching training. Dev. Sci..

[42] Buitenweg J.I.V., van de Ven R.M., Prinssen S., Murre J.M.J., Ridderinkhof K.R. (2017). Cognitive Flexibility Training: A Large-Scale Multimodal Adaptive Active-Control Intervention Study in Healthy Older Adults. Front. Hum. Neurosci..

[43] Goldstein J., Cajko L., Oosterbroek M., Michielsen M., Van Houten O., Salverda F. (1997). Video games and the elderly. Soc. Behav. Personal. Int. J..

[44] Basak C., Boot W.R., Voss M.W., Kramer A.F. (2008). Can training in a real-time strategy video game attenuate cognitive decline in older adults?. Psychol. Aging.

[45] Rosenberg D., Depp C.A., Vahia I.V., Reichstadt J., Palmer B.W., Kerr J., Norman G., Jeste D.V. (2010). Exergames for subsyndromal depression in older adults: A pilot study of a novel intervention. Am. J. Geriatr. Psychiatry.

[46] Brem M.H., Lehrl S., Rein A.K., Massute S., Schulz-Drost S., Gelse K., Schlechtweg P.M., Hennig F.F., Olk A., Jacob H.J. (2010). Stop of loss of cognitive performance during rehabilitation after total hip arthroplasty-prospective controlled study. J. Rehabil. Res. Dev..

[47] Gamberini L., Cardullo S., Seraglia B., Bordin A. (2010). Neuropsychological testing through a Nintendo Wii console. Stud. Health Technol. Inform..

[48] Peretz C., Korczyn A.D., Shatil E., Aharonson V., Birnboim S., Giladi N. (2011). Computer-based, personalized cognitive training versus classical computer games: A randomized double-blind prospective trial of cognitive stimulation. Neuroepidemiology.

[49] Maillot P., Perrot A., Hartley A. (2012). Effects of interactive physical-activity video-game training on physical and cognitive function in older adults. Psychol. Aging.

[50] Belchior P., Yam A., Thomas K.R., Bavelier D., Ball K.K., Mann W.C., Marsiske M. (2019). Computer and Videogame Interventions for Older Adults’ Cognitive and Everyday Functioning. Games Health J..

[51] Taylor B., Yam A., Belchior P., Marsiske M. (2021). Videogame and Computer Intervention Effects on Older Adults’ Mental Rotation Performance. Games Health J..

[52] Ramnath U., Rauch L., Lambert E.V., Kolbe-Alexander T. (2021). Efficacy of interactive video gaming in older adults with memory complaints: A cluster-randomized exercise intervention. PLoS ONE.

[53] Bacha J.M.R., Gomes G.C.V., de Freitas T.B., Viveiro L.A.P., da Silva K.G., Bueno G.C., Varise E.M., Torriani-Pasin C., Alonso A.C., Luna N.M.S. (2018). Effects of Kinect Adventures Games Versus Conventional Physical Therapy on Postural Control in Elderly People: A Randomized Controlled Trial. Games Health J..

[54] Chuang L.Y., Hung H.Y., Huang C.J., Chang Y.K., Hung T.M. (2015). A 3-month intervention of Dance Dance Revolution improves interference control in elderly females: A preliminary investigation. Exp. Brain Res..

[55] Guimaraes A.V., Barbosa A.R., Meneghini V. (2018). Active videogame-based physical activity vs. Aerobic exercise and cognitive performance in older adults: A randomized controlled trial. J. Phys. Educ. Sport.

[56] Manser P., de Bruin E.D. (2021). Making the Best Out of IT: Design and Development of Exergames for Older Adults With Mild Neurocognitive Disorder—A Methodological Paper. Front. Aging Neurosci..

[57] Torres A. Cognitive effects of videogames on older people. Proceedings of the 7th ICDVRAT with ArtAbilitation.

[58] Yamaguchi H., Maki Y., Takahashi K. (2011). Rehabilitation for dementia using enjoyable video-sports games. Int. Psychogeriatr..

[59] Hughes T.F., Flatt J.D., Fu B., Butters M.A., Chang C.C., Ganguli M. (2014). Interactive video gaming compared with health education in older adults with mild cognitive impairment: A feasibility study. Int. J. Geriatr. Psychiatry.

[60] Amjad I., Toor H., Niazi I.K., Pervaiz S., Jochumsen M., Shafique M., Haavik H., Ahmed T. (2019). Xbox 360 Kinect Cognitive Games Improve Slowness, Complexity of EEG, and Cognitive Functions in Subjects with Mild Cognitive Impairment: A Randomized Control Trial. Games Health J..

[61] Jahouh M., González-Bernal J.J., González-Santos J., Fernández-Lázaro D., Soto-Cámara R., Mielgo-Ayuso J. (2021). Impact of an Intervention with Wii Video Games on the Autonomy of Activities of Daily Living and Psychological-Cognitive Components in the Institutionalized Elderly. Int. J. Environ. Res. Public Health.

[62] Lin Y.F., Liu M.F., Ho M.H., Lin Y.K., Hsiao Y.L., Wang M.H., Chang C.C., Montayre J. (2022). A Pilot Study of Interactive-Video Games in People with Mild Cognitive Impairment. Int. J. Environ. Res. Public Health.

[63] Sato K., Ochi A., Watanabe K., Yamada K. (2023). Effects of dance video game training on cognitive functions of community-dwelling older adults with mild cognitive impairment. Aging Clin. Exp. Res..

[64] Rubenstein L.Z. (2006). Falls in older people: Epidemiology, risk factors and strategies for prevention. Age Ageing.

[65] Yeşilyaprak S.S., Yıldırım M.Ş., Tomruk M., Ertekin Ö., Algun Z.C. (2016). Comparison of the effects of virtual reality-based balance exercises and conventional exercises on balance and fall risk in older adults living in nursing homes in Turkey. Physiother. Theory Pract..

[66] Maki B.E., Sibley K.M., Jaglal S.B., Bayley M., Brooks D., Fernie G.R., Flint A.J., Gage W., Liu B.A., McIlroy W.E. (2011). Reducing fall risk by improving balance control: Development, evaluation, and knowledge-translation of new approaches. J. Saf. Res..

[67] Ambrose A.F., Paul G., Hausdorff J.M. (2013). Risk factors for falls among older adults: A review of the literature. Maturitas.

[68] de Bruin E., Dörflinger M., Reith A., Murer K. (2010). The effect of dance dance revolution gaming compared to conventional physical training on dual task walking costs in elderly. Park. Relat. Disord..

[69] William B., Doherty N.L., Bender A., Mattox H., Tibbs J.R. (2011). The effect of nintendo wii on balance: A pilot study supporting the use of the wii in occupational therapy for the well elderly. Occup. Ther. Health Care.

[70] Picchierri G., Murer K., de Bruin E.D. (2012). A cognitive-motor intervention using a dance video game to enhance foot placement accuracy and gait under dual task conditions in older adults: A randomized controlled trial. BMC Geriatr..

[71] Schoene D., Lord S.R., Delbaere K., Severino C., Davies T.A., Smith S.T. (2013). A randomized controlled pilot study of home-based step training in older people using videogame technology. PLoS ONE.

[72] Nicholson V.P., McKean M., Lowe J., Fawcett C., Burkett B. (2015). Six weeks of unsupervised Nintendo Wii Fit gaming is effective at improving balance in independent older adults. J. Aging Phys. Act..

[73] Kliem A., Wiemeyer J. (2010). Comparison of a traditional and a video game based balance training program. Int. J. Comput. Sci. Sport.

[74] Cui X., Lyness J.M., Tang W., Tu X., Conwell Y. (2008). Outcomes and predictors of late-life depression trajectories in older primary care patients. Am. J. Geriatr. Psychiatry.

[75] Vahia I.V., Meeks T.W., Thompson W.K., Depp C.A., Zisook S., Allison M., Judd L.L., Jeste D.V. (2009). Subsyndromal depression and successful aging in older women. Am. J. Geriatr. Psychiatry.

[76] Charney D.S., Reynolds C.F., Lewis L., Lebowitz B.D., Sunderland T., Alexopoulos G.S., Blazer D.G., Katz I.R., Meyers B.S., Arean P.A. (2003). Depression and Bipolar Support Alliance consensus statement on the unmet needs in diagnosis and treatment of mood disorders in late life. Arch. Gen. Psychiatry.

[77] Barbour K.A., Blumenthal J.A. (2005). Exercise training and depression in older adults. Neurobiol. Aging.

[78] Drazich B.F., LaFave S., Crane B.M., Szanton S.L., Carlson M.C., Budhathoki C., Taylor J.L. (2020). Exergames and Depressive Symptoms in Older Adults: A Systematic Review. Games Health J..

[79] Li J., Theng Y.L., Foo S. (2016). Exergames for Older Adults with Subthreshold Depression: Does Higher Playfulness Lead to Better Improvement in Depression?. Games Health J..

[80] Yang J.E., Lee T.Y., Kim J.K. (2017). The effect of a VR exercise program on falls and depression in the elderly with mild depression in the local community. J. Phys. Ther. Sci..

[81] Rodrigues E.V., Gallo L.H., Guimarães A.T.B., Melo Filho J., Luna B.C., Gomes A.R.S. (2018). Effects of Dance Exergaming on Depressive Symptoms, Fear of Falling, and Musculoskeletal Function in Fallers and Nonfallers Community-Dwelling Older Women. Rejuvenation Res..

[82] Heinbach M., Block A., Hubbard E., Cataldo J., Cooper B., Leutwyler H. (2021). Impact of exergames on psychiatric symptoms in older adults with serious mental illness. Aging Ment. Health.

[83] De Lima B.E., Passos G.S., Youngstedt S.D., Júnior L.C.B.S., Santana M.G. (2021). Effects of Xbox Kinect exercise training on sleep quality, anxiety and functional capacity in older adults. J. Bodyw. Mov. Ther..

[84] Otero P., Cotardo T., Blanco V., Vázquez F.L. (2021). Development of a Videogame for the Promotion of Active Aging Through Depression Prevention, Healthy Lifestyle Habits, and Cognitive Stimulation for Middle-to-Older Aged Adults. Games Health J..

